# THE INJURY RESISTANT ABILITY OF MELANOPSIN-EXPRESSING INTRINSICALLY PHOTOSENSITIVE RETINAL GANGLION CELLS

**DOI:** 10.1016/j.neuroscience.2014.11.002

**Published:** 2014-11-10

**Authors:** Q. Cui, C. Ren, P. J. Sollars, G. E. Pickard, K.-F. So

**Affiliations:** a Guangdong-HongKong-Macau Institute of CNS Regeneration, Jinan University, Guangdong, PR China; b Guangdong Medical Key Laboratory of Brain Function and Diseases, Jinan University, Guangzhou, PR China; c GHM Collaboration and Innovation Center for Tissue Regeneration and Repair, Jinan University, Guangzhou, PR China; d Department of Ophthalmology, University of Hong Kong, Hong Kong; e School of Veterinary Medicine and Biomedical Sciences, University of Nebraska, Lincoln, NE 68583, USA; f Department of Ophthalmology and Visual Sciences, University of Nebraska Medical Center, Omaha, NE 68198, USA

**Keywords:** melanopsin, intrinsically photosensitive retinal ganglion cell, injury, survival

## Abstract

Neurons in the mammalian retina expressing the photopigment melanopsin have been identified as a class of intrinsically photosensitive retinal ganglion cells (ipRGCs). This discovery more than a decade ago has opened up an exciting new field of retinal research, and following the initial identification of photosensitive ganglion cells, several subtypes have been described. A number of studies have shown that ipRGCs subserve photoentrainment of circadian rhythms. They also influence other non-image forming functions of the visual system, such as the pupillary light reflex, sleep, cognition, mood, light aversion and development of the retina. These novel photosensitive neurons also influence form vision by contributing to contrast detection. Furthermore, studies have shown that ipRGCs are more injury-resistant following optic nerve injury, in animal models of glaucoma, and in patients with mitochondrial optic neuropathies, i.e., Leber’s hereditary optic neuropathy and dominant optic atrophy. There is also an indication that these cells may be resistant to glutamate-induced excitotoxicity. Herein we provide an overview of ipRGCs and discuss the injury-resistant character of these neurons under certain pathological and experimental conditions.

## INTRODUCTION

Rods and cones were long believed to be the only mammalian photo-sensitive cells. Phototransduction signals from these cells propagate through the retinal circuitry to modulate action potential firing in retinal ganglion cells (RGCs), the projection neurons of the retina. RGCs send the light information via their axons, which constitute the optic nerve, to targets in the brain, such as the lateral geniculate nucleus (LGN) of the thalamus, the midbrain superior colliculus (SC), and the hypothalamic suprachiasmatic nucleus (SCN) which mediate form vision, orienting and avoidance responses, and photoentrainment of circadian rhythms, respectively. The LGN relays light information further to the visual cortex for the complex processing necessary for visual perception (Pickard and Sollars, 2012).

In the 1980s, data began accumulating to suggest that circadian rhythms generated by the SCN circadian clock could be shifted in mice in which virtually all classic photoreceptors had degenerated. Moreover, the spectral sensitivity of the photoreceptor that produced these behavioral phase shifts was unlike that of either rods or cones (Foster et al., 1991; Provencio et al., 1994; Yoshimura and Ebihara, 1996). Importantly, the light-induced effects on SCN-clock function in rodless/coneless mice still required illumination of the eyes (Freedman et al., 1999; Lucas et al., 1999). These findings offered the possibility that not all light signals transmitted from the retina to the brain begin with the rod and cone photoreceptors in the eye. This was further supported by observations that: (1) light remained effective in suppressing pineal melatonin secretion and entraining the circadian rhythms in blind patients with severe loss of rods and cones (Czeisler et al., 1995), and (2) photic activation of the SCN was found in newborn mice which had yet to complete development of the retinal rod and cone circuitry (Weaver and Reppert, 1995). Collectively, these reports raised the otherwise unanticipated possibility of the existence of a third photoreceptor in the mammalian eye.

### Intrinsically photosensitive RGCs (ipRGCs)

The above-mentioned observations motivated the search for novel photopigments in the mammalian retina. Provencio and his colleagues identified a new opsin, termed melanopsin that was expressed in both primate and rodent retinas but in RGCs rather than rods or cones (Provencio et al., 1998, 2000). Shortly thereafter a vitamin A-based photopigment with peak sensitivity to ~480-nm light was identified functionally in rodless/coneless mice using the pupillary light reflex as a behavioral response (Lucas et al., 2001). The search culminated in 2002 with the breakthrough discovery of ipRGCs (Berson et al., 2002; Hattar et al., 2002). Berson and his colleagues (2002) found that SCN-projecting RGCs responded to light by depolarizing and increasing their firing rate. Electrophysiological analyses showed that these SCN-projecting RGCs respond to light after pharmacological blockade of all signals from the rods and cones, and even after ipRGCs were physically isolated from the rest of the retina. It was concluded that RGCs projecting to the SCN were bona fide photoreceptors, and that these unconventional RGCs probably expressed the recently identified melanopsin protein. Hattar and coworkers (2002) confirmed these observations by showing that indeed these light-responsive RGCs expressed melanopsin and were afferent to the SCN. Additional studies confirmed that melanopsin was the photopigment responsible for bestowing photosensitivity to these RGCs (Panda et al., 2002; Ruby et al., 2002; Lucas et al., 2003). It is now widely acknowledged that, in adult mammals, melanopsin is expressed only in ipRGCs, not in other cell types, and melanopsin is distributed throughout the plasma membrane of both the somata and their dendrites (Belenky et al., 2003; Do et al., 2009).

### Multiple types of ipRGC

**Fig. 1:**
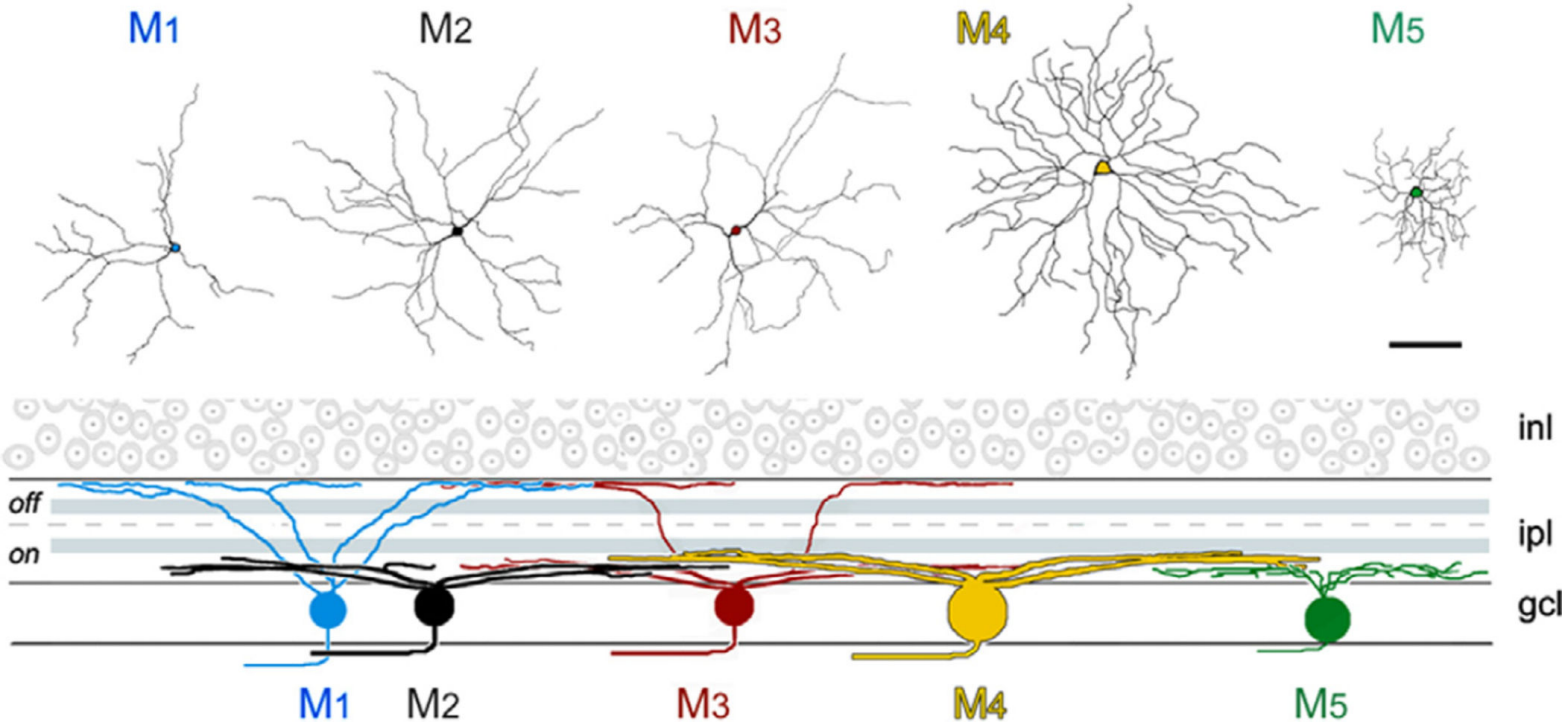
Morphology of five types of intrinsically photosensitive retinal ganglion cell (ipRGC). Top: *en face* view (scale bar=100 µm). Bottom: Dendritic stratification as viewed in schematic radial section. Pale blue bands in the inner plexiform layer (IPL) are the ON and OFF cholinergic bands. There are two bands of melanopsin dendrites, both outside the cholinergic bands. One lies at the margin of the inner nuclear layer (INL), and the second, broader band sits close to the ganglion cell layer (GLC). The outer band contains processes of M1 and M3 cells, the inner one the processes of M2, M3, M4, and M5 cells. There are subtle differences in stratification among the inner-stratifying population. Image from ‘Intrinsically photosensitive retinal ganglion cells’, Berson DM, reprinted courtesy of The MIT Press from *The New Visual Neurosciences* edited by John S. Werner and Leo M. Chalupa.

Initially identified as a single type of RGC (Berson et al., 2002; Hattar et al., 2002), additional morphological and physiological studies have revealed that ipRGCs comprise a far more complex population than originally thought. Based on their morphology, molecular markers, retinofugal projections, intrinsic photosensitivity, melanopsin protein level, and other electrophysiological properties, ipRGCs are at present believed to comprise at least six types, namely M1–M6 (Fig. 1) (Sekaran et al., 2003; Tu et al., 2005; Jusuf et al., 2007; Viney et al., 2007; Baver et al., 2008; Schmidt et al., 2008, 2014; Badea et al., 2009; Schmidt and Kofuji, 2009; Ecker et al., 2010; Pérez de Sevilla Müller et al., 2010; Estevez et al., 2012; Sand et al., 2012; Zhao et al., 2014), with M6 being recently identified (Quattrochi et al., 2013).

The best-characterized ipRGCs are the M1, M2 and M3 types. The majority of M1 cells are located in the ganglion cell layer (GCL) (with some displaced to the inner nuclear layer) and these cells constitute only about 1% (700–900 overall) of the mouse RGC population, but their ~300-µm diameter dendritic fields tile the entire retinal surface (Berson et al., 2010). The most distinguishable feature among ipRGC subtypes is the region in which their dendrites stratify in the inner plexiform layer (IPL) (Fig. 1). M1 cell dendrites stratify at the outermost margin of the IPL, at the border with the inner nuclear layer (INL) (for review, see Schmidt et al., 2011). This is the classic physiologic “OFF-sublamina” of the IPL where OFF-bipolar cells distribute their axon terminals. Despite their dendrites terminating in the OFF-sub-lamina, M1 cells receive synaptic input from ON-bipolar cells in what has been termed an accessory ON-layer (Dumitrescu et al., 2009; Hoshi et al., 2009). M1 cells have a noticeably high level of melanopsin immunoreactivity (Hattar et al., 2006; Baver et al., 2008). Consequently, M1 cells show the highest intrinsic photosensitivity among the ipRGC types and they also produce the largest intrinsic photocurrent (Schmidt and Kofuji, 2009; for review, see Do and Yau, 2010). A subset of ipRGCs, most likely M1 cells, has intraretinal collateral axonal branches that terminate in the IPL (Joo et al., 2013). These collateral branches are probably responsible for the light-driven responses of dopaminergic amacrine cells that exhibit sustained melanops-independent light responses (Zhang et al., 2008, 2012). Unexpectedly, M1 ipRGCs have recently been described to send axons into the iris and ciliary body where they appear to participate in the pupillary light reflex (Schmidt et al., 2013; Semo et al., 2014).

Compared with M1 ipRGCs, M2 ipRGCs have larger somata and a more complex dendritic arbor (Hattar et al., 2006; Schmidt and Kofuji, 2009; Berson et al., 2010). The number of M2 cells is similar to M1 cells and M2 ipRGCs also tile the entire retina (Hattar et al., 2006; Berson et al., 2010). Importantly, the dendrites of M2 ipRGCs stratify in the ON-sublamina of the IPL near the border with the GCL (Hattar et al., 2006; Baver et al., 2008; Schmidt and Kofuji, 2009). M2 ipRGCs have an intrinsic photosensitivity that is less than the intrinsic photosensitivity of M1 ipRGCs and they produce a 10-fold smaller maximum photocurrent (Schmidt and Kofuji, 2009). However, they can fire action potentials at far higher frequencies than the M1 cells (Schmidt and Kofuji, 2009). Thus, synaptic input may be more important for driving the M2 ipRGCs over their full dynamic range than it is for driving the M1 cells (for review, see Do and Yau, 2010).

The dendrites of M3 ipRGCs bistratify in both the inner ON and outer OFF-sublaminae of the IPL, and comprise less than 10% of the ipRGCs (Berson et al., 2010; Schmidt et al., 2011). Detailed analyses of the M3 ipRGCs have revealed that these bistratified RGCs, in contrast to other bistratified RGCs, show variability in the proportion of dendritic stratification in the ON and OFF sublaminae and their dendritic fields do not cover the entire retina (Schmidt and Kofuji, 2011). This has led to questioning whether these RGCs actually represent a specific type of ipRGC (Berson et al., 2010). The M3 ipRGCs are otherwise similar to M2 cells in the size and complexity of their dendritic arbors (Schmidt and Kofuji, 2011). All other ipRGC types including M3 cells are less intrinsically photosensitive than M1 ipRGCs (Schmidt and Kofuji, 2009, 2011; Ecker et al., 2010). This variation in intrinsic photosensitivity may be associated with the different levels of melanopsin in these ipRGC types, because compared to M1 cells, melanopsin abundance appears to be lower in the M2 cells and even lower in the M3 ipRGCs (Schmidt and Kofuji, 2009; Berson et al., 2010; Ecker et al., 2010; Estevez et al., 2012). Thus, ipRGCs as a class may tune their intrinsic sensitivities by their level of melanopsin expression (for review, see Schmidt et al., 2011).

In a study using transgenic mice in which a green fluorescent protein labels melanopsin RGCs, two additional types of ipRGC, M4 and M5, were revealed (Ecker et al., 2010). Both of these ipRGC types stratify in the ON sublamina of the IPL, but each has a unique morphology and can be differentiated from M2 cells (Fig. 1). M4 cells have the largest soma of any described ipRGC subtype, as well as larger and even more complex dendritic arbors than M2 cells (Ecker et al., 2010). By contrast, M5 ipRGCs have small, highly branched arbors arrayed uniformly around the soma (Ecker et al., 2010; for review, see Schmidt et al., 2011). Owing to the low expression level of melanopsin, M4 and M5 subtypes can only be labeled with melanopsin antibody after immunostaining amplification techniques are performed. Consistent with very low levels of melanopsin, these cells exhibit a weak intrinsic light response (Ecker et al., 2010). Nevertheless, the melanopsin-driven intrinsic photo-response of M4 ipRGCs appears to play a functional role contributing to visual contrast sensitivity and also allowing these cells to signal prior light exposure and environmental luminance over long periods of time (Schmidt et al., 2014). Preliminary observations from a transgenic mouse line in which the Cadherin-3 promoter drives enhanced green fluorescent protein (EGFP) expression, have identified a new ipRGC tentatively referred to as M6. These cells have spiny, densely branched dendritic arbors that often stratify in two sub-laminae of the IPL, express very low levels of melanopsin and produce small intrinsic light responses similar to M4 and M5 ipRGCs (Quattrochi et al., 2013). Although the function of M6 ipRGCs remains to be determined, their projections overlap with those of other ipRGCs, terminating in the olivary pretectal nucleus (OPN) and the intergeniculate leaflet (IGL) (Quattrochi et al., 2013).

**Fig. 2:**
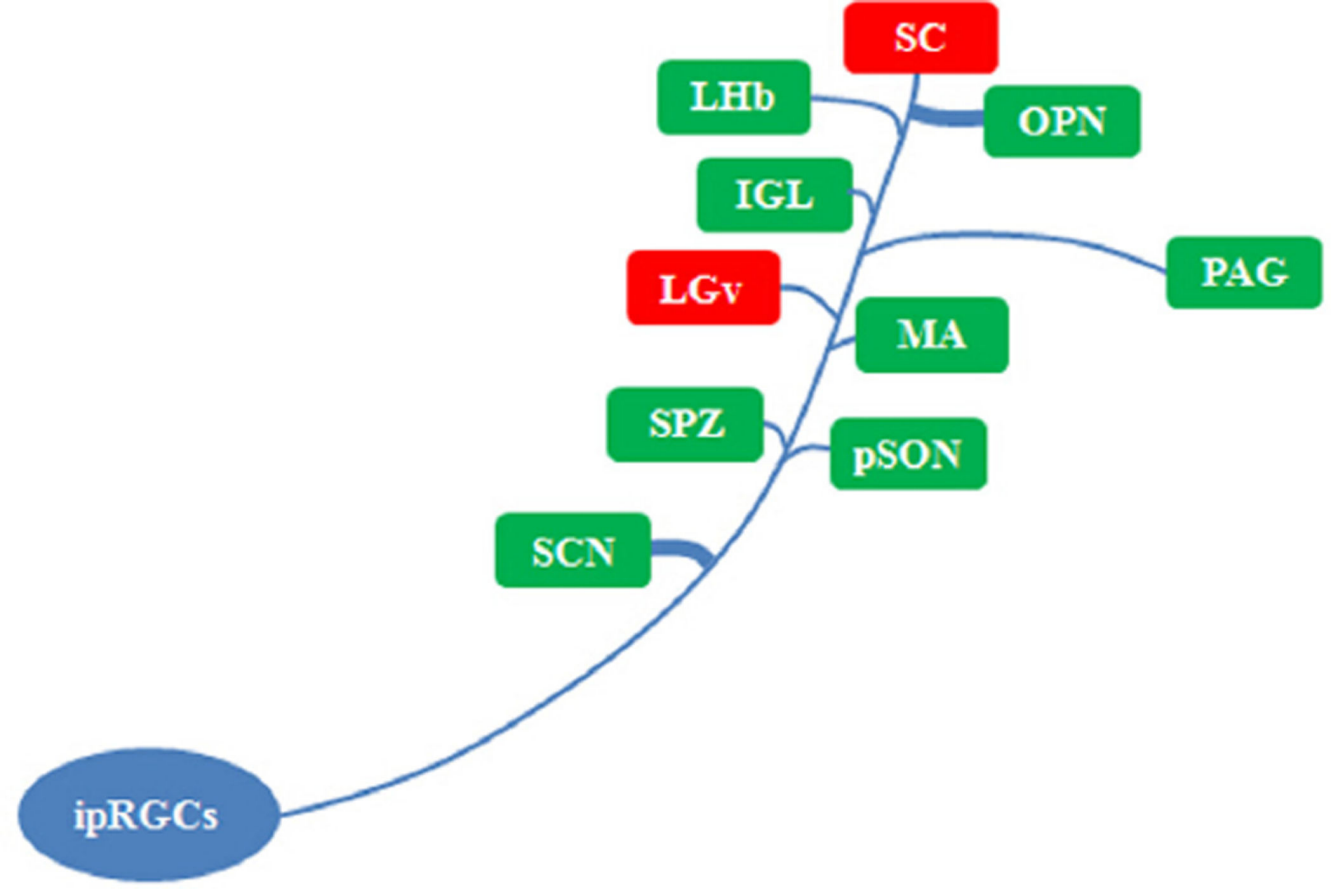
Central targets for intrinsically photosensitive retinal ganglion cells (ipRGCs). Visual-related nuclei are shown in red and nonimaging centers are shown in green. The illustration is descriptive as ipRGC projections are not uniform in density. Abbreviations (functions involved): SCN, suprachiasmatic nucleus (circadian rhythms); SC, superior colliculus (vision and eye movement); LGv, lateral geniculate nucleus, ventral division (visuomotor functions); IGL, intergeniculate leaflet (circadian rhythms); OPN, olivary pretectal nucleus (pupillary light reflex); PAG, the rostral periaqueductal gray (conditioned fear, pain and analgesia); MA, medial amygdaloid nucleus (reproductive behavior and conditioned fear); SPZ, subparaventricular zone (sleep and wake regulation); pSON, peri-supraoptic nucleus (neuroendocrine output); LHb, lateral habenula (reward processing, pain and reproductive behavior).

The discovery of ipRGCs provided the final proof that some light responses in mammals could originate with non-rod, non-cone photoreceptors in the retina. It also represented a breakthrough in our understanding of the retinal circuitry responsible for a number of biological functions. The past decade has seen this fundamental discovery expand in a number of important directions (Lucas, 2013). Briefly, it is now clear that ipRGCs target numerous discrete brain regions involved in both non-image-forming and image-forming vision (Pickard, 1985; Morin et al., 2003; Hattar et al., 2006; Fig. 2). In addition to their critical role in mediating circadian photoentrainment, ipRGCs also contribute signals regulating the pupillary light reflex and influencing sleep (Berson et al., 2002; Göz et al., 2008; Güler et al., 2008; Hatori et al., 2008; Tsai et al., 2009; Altimus et al., 2010). During late gestation ipRGCs are responsible for mediating the effects of light on retinal development (Rao et al., 2013). During the early neonatal period ipRGCs are responsible for behavioral responses to light (i.e., negative phototaxis) before the rod/cone circuitry is fully developed (Johnson et al., 2010). Notable central targets of ipRGCs are the SCN and the IGL for circadian entrainment, the OPN for the pupillary light reflex, the ventral subparaventricular zone (vSPZ) for masking behavior, the ventrolateral preoptic nucleus (VLPO) for sleep, and the dorsal lateral geniculate nucleus (dLGN) for image formation (Hattar et al., 2002, 2006; Gooley et al., 2003; Hannibal and Fahrenkrug, 2004; Barnard et al., 2006; Brown et al., 2010; Ecker et al., 2010; Matynia, 2013; Schmidt et al., 2014). As is common for conventional RGCs, ipRGCs also send axon collaterals to innervate multiple brain regions (Pickard, 1985; Morin et al., 2003; Hattar et al., 2006).

In addition to ipRGCs, conventional RGCs also innervate these same targets, with proportions that vary across brain regions and species. For instance, virtually all retinal innervation of the mouse SCN is from ipRGCs (Hattar et al., 2006; Baver et al., 2008; Güler et al., 2008), whereas in the golden hamster, the ipRGCs constitute 80–90% (Sollars et al., 2003). It is possible that these varying proportions of inputs from ipRGCs and conventional RGCs correspond to some differences in nonimage vision across species. In addition, conventional RGCs projecting to non-image forming brain regions may also regulate certain biological functions. It was recently shown that both ON and OFF Y-like RGCs with alpha RGC morphology innervate the dorsal raphe nucleus (DRN) in the Mongolian gerbil (Luan et al., 2011), and that these RGCs influence non-vision functions such as serotonergic tone and mood (Ren et al., 2013). Although ON-alpha (M4) RGCs do express low levels of melanopsin and are intrinsically photosensitive (Estevez et al., 2012; Schmidt et al., 2014), no melanopsin was detected in DRN-projecting RGCs which also includes OFF-alpha cells (Luan et al., 2011). Recently, we characterized an RGC population that projects to the caudal periaqueductal gray (cPAG) in the Mongolian gerbil (Ren et al., 2014). In the mouse, a weak ipRGC projection has been described to the region of the PAG (Hattar et al., 2006). However, the function of cPAG-projecting RGCs, as well as those RGCs projecting to additional brain regions, remains to be determined.

### ipRGCs are resistant to injury

ipRGCs are atypical central nervous system neurons, acting both as photoreceptors responding directly to environmental stimuli and as standard neurons integrating synaptic input and generating action potentials even in the absence of intrinsic phototransduction (Berson, 2003; Pickard et al., 2009). These properties, in and of themselves, would not necessarily have predicted ipRGCs to be resistant to traumatic injury or to be protected from certain pathological conditions. However, a growing literature indicates that indeed ipRGCs are less vulnerable to damage and disease compared to conventional RGCs. Owing to the labeling technique used, the work on injury resistant ability of ipRGCs is mainly focused on M1 type.

### ipRGCs are resistant to optic nerve damage

It has been known for several decades that a small percentage of RGCs survive for protracted periods following optic nerve transection (Holländer et al., 1985). The first example of ipRGCs’ enhanced survival properties came from a study examining RGC persistence following optic nerve transection in the mouse. Melanopsin-immunopositive RGCs (most likely M1 ipRGCs) showed a threefold increase in survival rate compared to non-melanopsin RGCs when examined 1 month after optic nerve injury (Robinson and Madison, 2004). Similarly, melanopsin-immunopositive RGC enhanced survival was observed 2 weeks after optic nerve injury in rats (Li et al., 2008). More recently it was reported that the M1 ipRGC is the most common RGC type that remains after rat optic nerve transection, comprising 82% of surviving RGCs 60 days after injury (Pérez de Sevilla Müller et al., 2014). Despite this enhanced survival after injury to their axons, ipRGCs did not show increased axonal regrowth into nerve grafts compared to conventional RGCs, suggesting that the mechanisms underlying ipRGCs’ ability to survive following axonal injury differs from the cellular mechanisms promoting regrowth of their injured axons (Robinson and Madison, 2004). Similar findings were also obtained in an optic nerve crush model in which ipRGCs showed enhanced survival but not enhanced axon regeneration into the distal part of the crushed optic nerve (K Park, personal communication).

The cellular/molecular mechanisms underlying the survival of ipRGCs following optic nerve transection are currently unknown. However, one factor that may contribute to the survival of M1 ipRGCs after optic nerve damage is the undamaged axon collateral that remains within the eye (Joo et al., 2013; Schmidt et al., 2013; Semo et al., 2014). These ipRGCs may derive trophic support from within the retina for enhanced survival after optic nerve injury. Although the exact number of M1 ipRGCs that send collaterals into the retina, iris and ciliary marginal zone (Semo et al., 2014) is not known, this specific subset of ipRGCs may be too small to fully account for the increased survival observed after optic nerve damage. Among the possible mechanisms that may contribute to ipRGCs’ superior survival after damage is melanopsin phototransduction. At present it is not known whether melanopsin-mediated phototransduction contributes to M1 ipRGC survival after optic nerve transection. This could be examined using reporter knock-in mouse models in which a reporter gene replaces the melanopsin (Opn4) gene allowing M1 ipRGCs to be identified in the absence of melanopsin protein (Pickard et al., 2009). Therefore, using mice homozygous for the reporter (Opn4^−/−^), ipRGC survival could be examined after optic nerve transection in the absence of melanopsin phototransduction.

### ipRGCs are resistant to damage in animal models of glaucoma

Glaucoma is an ocular disorder typically associated with raised intraocular pressure (IOP) resulting in optic nerve damage and the loss of RGCs, and several groups have examined the sparing of ipRGCs in rodent models of glaucoma. While conventional RGC number was decreased in rats with an increase in IOP produced by laser cauterization, no change in the number of melanopsin-containing RGCs was seen (Li et al., 2006), suggesting that ipRGCs are resistant to the deleterious effects produced by IOP elevation. However, in CFP-D2 mice that develop a naturally occurring elevation of IOP with increasing age, ipRGCs appeared to be resistant to damage resulting from IOP elevation at an early age (5 months old), but became vulnerable at a later age (11 months old) (Zhang et al., 2013). This discrepancy may be related to the magnitude of IOP changes observed at the different ages. While IOP was significantly increased from 2 to 5 months of age, IOP increased even further at 11-months (Zhang et al., 2013). Thus the IOP threshold for inducing damage to ipRGCs may be significantly higher than the IOP levels that induce damage to conventional RGCs. It should be noted that in other rodent models of glaucoma, ipRGCs appeared to be vulnerable to damage to an extent similar to that of conventional RGCs (Drouyer et al., 2008; de Zavalia et al., 2011).

ipRGC activity has begun to be examined in patients with glaucoma using either light-induced reduction nocturnal pineal melatonin secretion or the pupillary light reflex as functional readouts of the melanopsin-based phototransduction. In most reports the results indicate significant reduction in ipRGC function in the affected eye compared either to the unaffected eye or to normal populations (Pérez-Rico et al., 2010; Kankipati et al., 2011; Nissen et al., 2014). However, in the case study of a glaucoma patient who had no light perception vision and marked retinal nerve fiber layer loss in the affected eye, a minimal pupillary light reflex was observed (Zhou et al., 2014), suggesting some sparing of ipRGC function.

The cellular/molecular mechanisms that appear to protect ipRGCs in animal models of glaucoma are not understood and again, it is not known whether melanopsin-based intrinsic photosensitivity plays a role. One difference between the results obtained in animal models of glaucoma and glaucoma patients is the endpoint examined: i.e., morphological vs functional. Future studies examining ipRGCs in animal models of glaucoma should examine both melanopsin immunoreactive RGCs and an ipRGC functional measure such as photoentrainment or the pupillary light reflex (see Drouyer et al., 2008). Moreover, ipRGC type must also be considered since ipRGCs differentially innervate their central targets (Baver et al., 2008) and thus one behavioral endpoint might be significantly altered whereas another might be less affected.

### ipRGCs and inherited optic neuropathies

Hereditary optic neuropathies are a group of disorders with prominent optic nerve degeneration and dysfunction. The most common of these disorders are dominant optic neuropathy or atrophy (Kjers’ disease) and Leber’s hereditary optic neuropathy (LHON). These diseases are associated with mutations in mitochondrial DNA, although the exact mechanisms of mitochondrial impairment have yet to be determined. Due to high metabolic demands, it has been suggested that the optic nerve may be particularly vulnerable to perturbations in mitochondrial function (Bristow et al., 2002; Newman, 2005). In patients with mitochondrial optic neuropathies, i.e., LHON and dominant optic atrophy (DOA), and severe vision loss, ipRGC function was tested by examining the light-induced suppression of nocturnal melatonin secretion (mediated via ipRGC input to the SCN). Light-induced melatonin suppression in LHON and DOA patients was maintained as in controls, indicating that the retinohypothalamic tract is sufficiently preserved in these patients to stimulate the descending autonomic circuits to the pineal. Importantly, histological investigation of post-mortem eyes from two LHON patients and one with DOA, revealed that melanopsin-containing RGCs were relatively spared compared with the massive loss of total RGCs (La Morgia et al., 2010). Using the pupillary light reflex to assess preservation of ipRGCs in LHON, Moura and colleagues also reached the conclusion that there was a selective preservation of ipRGCs (Moura et al., 2013). Similar sparing of the pupillary light reflex was observed in a group of patients with hereditary optic neuropathy with optic nerve atrophy and vision loss (Kawasaki et al., 2014). These observations suggest that ipRGCs resist neurodegeneration caused by mitochondrial dysfunction and maintain non-image-forming functions of the eye in these highly visually impaired patients.

Mutations in the optic atrophy 1 (*OPA1*) gene are commonly associated with DOA patients. The product of the OPA1 gene, a dynamin-related guanosine triphosphatase, is targeted to the mitochondrial inner membrane and may play a role in the stabilization of mitochondrial membrane integrity (Newman, 2005). Mouse models have been used to examine the role of OPA1 in RGC function and the pathophysiology of vision loss (Williams et al., 2011). Using the B6;C3-Opa1^Q285STOP^ mouse model, Perganta and colleagues reported that ipRGC morphology and function were completely preserved, supporting the clinical observations that ipRGCs are protected in mitochondrial optic neuropathies (Perganta et al., 2013). It is not known why ipRGCs are less susceptible to these devastating mitochondrial diseases of the retina. It has been suggested that these cells may have a reduced energy demand and therefore be less vulnerable to mitochondrial dysfunction although, conversely, it has been shown ipRGCs have an unusually high accumulation of mitochondria in their dendrites (Belenky et al., 2003). ipRGC morphology and function have also been examined in animal models of diabetic retinopathy. Morphological changes and reduced ipRGC function have been consistently described although the total number of ipRGCs is not reduced (Gastinger et al., 2008; Kumar and Zhou, 2011; Lahouaoui et al., 2014).

### ipRGCs are protected from N-methyl-d-aspartate (NMDA)-induced excitotoxicity

Following neuronal injury or under pathological conditions that lead to the excessive release of the neurotransmitter glutamate, a complex cascade of events occurs, leading to calcium dysregulation and subsequent neuronal death i.e., glutamate-induced excitotoxicity (Lipton and Rosenberg, 1994). As in other central nervous system neurons, the glutamate-evoked rise in intracellular calcium [Ca^2+^]_i_ in conventional RGCs is predominately mediated by the NMDA-type glutamate receptor as NMDA application *in vivo* and *in vitro* kills RGCs (Siliprandi et al., 1992; Lam et al., 1999; Li et al., 1999; Hartwick et al., 2008; but see also Ullian et al., 2004). ipRGCs receive glutamatergic input from bipolar cells, express AMPA, kainate and NMDA glutamate receptors, and are depolarized by glutamate (Belenky et al., 2003; Hartwick et al., 2007; Jakobs et al., 2007; Bramley et al., 2010). Thus, it might appear that ipRGCs would also be susceptible to the damaging effects of excess glutamate.

However, before the discovery of ipRGCs it was known that SCN-projecting RGCs were resistant to glutamate-induced toxicity; the retinohypothalamic tract was spared in rodents after peripheral glutamate injections whereas most RGCs projecting to the primary visual system were killed (Nemeroff et al., 1977; Pickard et al., 1982; Chambille, 1998). More recently these indirect findings that ipRGCs were less vulnerable to glutamate-induced toxicity were confirmed; in mice receiving intraocular NMDA injections, M1 ipRGCs were protected from the extensive cell death experienced by conventional RGCs (DeParis et al., 2012). It is currently not known why ipRGCs are resistant to NMDA excitotoxicity although the specific subunits that form the NMDA receptor in ipRGCs may play a role. Conventional NMDA receptors are typically comprised of GluN1 and GluN2 (previously NR1 and NR2) subunits to form a functional channel. However, they may also contain the modulatory GluN3A (previously NR3A) subunit that dramatically decreases the Ca^2+^ permeability of the NMDA receptor-associated channel. Cells expressing the GluN3A subunit display greater resistance to NMDA-mediated neurotoxicity (Nakanishi et al., 2009; Henson et al., 2010). It has been suggested that ipRGCs express the GluN3A receptor subunit, although GluN3A subunit expression is not unique to ipRGCs (Jakobs et al., 2007).

All of the studies to date on ipRGCs in injury models or under pathological conditions have examined M1 ipRGCs that express a high level of melanopsin and are thus easily identified. It is not clear whether melanopsin and intrinsic photosensitivity play a role in protecting RGCs from injury. This could be examined using knock-in mouse models in which a reporter replaces the Opn4 gene allowing M1 ipRGCs to be identified in the absence of melanopsin protein (Hattar et al., 2002; Pickard et al., 2009). The more sensitive Opn4-cre reporter mouse model (Ecker et al., 2010) could also be used to test whether other ipRGCs types that express much lower levels of melanopsin (M2–M6) are also resistant to optic nerve injury or NMDA-induced excitotoxicity. For example, in addition to M1 ipRGCs, ON alpha-RGCs were somewhat resistant to optic nerve transection in the rat (Pérez de Sevilla Müller et al., 2014) and these cells appear to be the M4 ipRGC subtype (Estevez et al., 2012; Schmidt et al., 2014).

It is unlikely that a single molecular/cellular mechanism is responsible for ipRGCs’ resistance to injury or disease. Intraretinal axon collaterals and NMDA receptor subunits with low Ca^2+^ permeability probably play some role in the survival of M1 ipRGCs following optic nerve damage and glutamate excitotoxicity, respectively. Molecular mechanisms that might contribute to ipRGC survival include the pathways for phosphatase and tensin homolog/mammalian target of rapamycin (PTEN/mTOR) and Janus kinase/signal transducer and activator of transcription (JAK/STAT). JAK/STAT proteins are activated in response to several cytokines and growth factors and mediate neuronal survival including RGCs (Huang et al., 2007). Whereas resistance to NMDA-induced excitotoxicity does not appear to depend on JAK/STAT signaling (DeParis et al., 2012), the PI3K/Akt cascade may play a role in ipRGC survival after optic nerve transection (Li et al., 2008). In the past, we have shown that JAK/STAT and PI3K/Akt signaling pathways are involved in ciliary neurotrophic factor and cAMP elevation- mediated RGC survival and axonal regeneration (Cui et al., 2003; Park et al., 2004).

Pituitary adenylate cyclase-activating polypeptide (PACAP) has been shown to have neuroprotective effects in the retina when administered intraocularly; when injected in low concentrations into the vitreous it protects conventional RGCs from glutamate-induced excitotoxicity (for review, see Atlasz et al., 2010). In the retina, PACAP is expressed exclusively in ipRGCs (Hannibal et al., 2002, 2014). Thus, PACAP may play a role in ipRGCs’ survival under conditions of glutamate-induced cell death, although whether neuronal expression of PACAP could mediate these effects is unknown. PACAP knockout mice could be used to examine the extent to which PACAP contributes to ipRGC resistance to injury and excitotoxicity (Reglodi et al., 2012).

## CONCLUSION

In adult mammals, melanopsin is expressed only in ipRGCs, and all ipRGCs appear to express melanopsin (Do and Yau, 2010). There are at least six types of melanopsin-containing ipRGCs, targeting various brain regions involved in both image-forming and non-image-forming functions. For unknown reasons, ipRGCs possess a higher intrinsic ability to survive under certain pathological and experimental conditions. A better understanding of the cellular and molecular mechanisms that provide neuroprotection to these RGCs may provide valuable insights for designing strategies to diminish the loss of vision following optic nerve injury or ocular disease.

## Abbreviations

cPAG: caudal periaqueductal gray
DOA: dominant optic atrophy
DRN: dorsal raphe nucleus
GCL: ganglion cell layer
IGL: intergeniculate leaflet
INL: inner nuclear layer
IOP: intraocular pressure
IPL: inner plexiform layer
ipRGCs: intrinsically photosensitive retinal ganglion cells
JAK/STAT: Janus kinase/signal transducer and activator of transcription
LGN: lateral geniculate nucleus
LHON: Leber’s hereditary optic neuropathy
NMDA: *N*-methyl-d-aspartate
OPN: olivary pretectal nucleus
PACAP: pituitary adenylate cyclase-activating polypeptide
RGCs: retinal ganglion cells
SCN: suprachiasmatic nucleus.
